# Supplementary material for: Unveiling the Bioactive Potential of the Invasive Jellyfish Phyllorhiza punctata Through Integrative Transcriptomic and Proteomic Analyses
Source: Biomolecules. 2025 Aug 4;15(8):1121. doi: 10.3390/biom15081121 (PMC12383608; doi:10.3390/biom15081121)
Supplement: Supplementary file 1 [file biomolecules-15-01121-s001.zip › Suplementary Material/FileS3/PEPFOLD4/XP_065052233_5261/clusters.html]

 PEP-FOLD clusters


Download archive of all models  here   

|  |  |  |
| --- | --- | --- |
| # | file | sOPEP |
| # cluster 1 - Size: 80 |  |  |
|  | XP\_065052233\_5261-model1 | -4.32902 |
|  | XP\_065052233\_5261-model1.1 | -4.32654 |
|  | XP\_065052233\_5261-model1.2 | -4.32654 |
|  | XP\_065052233\_5261-model1.3 | -4.32654 |
|  | XP\_065052233\_5261-model1.4 | -4.32087 |
|  | XP\_065052233\_5261-model1.5 | -4.32087 |
|  | XP\_065052233\_5261-model1.6 | -4.32087 |
|  | XP\_065052233\_5261-model1.7 | -4.32087 |
|  | XP\_065052233\_5261-model1.8 | -4.32087 |
|  | XP\_065052233\_5261-model1.9 | -4.32087 |
|  | XP\_065052233\_5261-model1.10 | -4.32087 |
|  | XP\_065052233\_5261-model1.11 | -4.32087 |
|  | XP\_065052233\_5261-model1.12 | -4.3186 |
|  | XP\_065052233\_5261-model1.13 | -4.3186 |
|  | XP\_065052233\_5261-model1.14 | -4.3186 |
|  | XP\_065052233\_5261-model1.15 | -4.3186 |
|  | XP\_065052233\_5261-model1.16 | -4.3186 |
|  | XP\_065052233\_5261-model1.17 | -4.3186 |
|  | XP\_065052233\_5261-model1.18 | -4.3186 |
|  | XP\_065052233\_5261-model1.19 | -4.3186 |
|  | XP\_065052233\_5261-model1.20 | -4.3186 |
|  | XP\_065052233\_5261-model1.21 | -4.3186 |
|  | XP\_065052233\_5261-model1.22 | -4.3186 |
|  | XP\_065052233\_5261-model1.23 | -4.3186 |
|  | XP\_065052233\_5261-model1.24 | -4.3186 |
|  | XP\_065052233\_5261-model1.25 | -4.3186 |
|  | XP\_065052233\_5261-model1.26 | -4.3186 |
|  | XP\_065052233\_5261-model1.27 | -4.3186 |
|  | XP\_065052233\_5261-model1.28 | -4.3186 |
|  | XP\_065052233\_5261-model1.29 | -4.3186 |
|  | XP\_065052233\_5261-model1.30 | -4.30107 |
|  | XP\_065052233\_5261-model1.31 | -4.30107 |
|  | XP\_065052233\_5261-model1.32 | -4.30107 |
|  | XP\_065052233\_5261-model1.33 | -4.30107 |
|  | XP\_065052233\_5261-model1.34 | -4.30107 |
|  | XP\_065052233\_5261-model1.35 | -4.30107 |
|  | XP\_065052233\_5261-model1.36 | -4.30107 |
|  | XP\_065052233\_5261-model1.37 | -4.30107 |
|  | XP\_065052233\_5261-model1.38 | -4.30107 |
|  | XP\_065052233\_5261-model1.39 | -4.29734 |
|  | XP\_065052233\_5261-model1.40 | -4.29734 |
|  | XP\_065052233\_5261-model1.41 | -4.29734 |
|  | XP\_065052233\_5261-model1.42 | -4.29562 |
|  | XP\_065052233\_5261-model1.43 | -4.29562 |
|  | XP\_065052233\_5261-model1.44 | -4.28788 |
|  | XP\_065052233\_5261-model1.45 | -4.28472 |
|  | XP\_065052233\_5261-model1.46 | -4.28472 |
|  | XP\_065052233\_5261-model1.47 | -4.28472 |
|  | XP\_065052233\_5261-model1.48 | -4.28472 |
|  | XP\_065052233\_5261-model1.49 | -4.28472 |
|  | XP\_065052233\_5261-model1.50 | -4.28472 |
|  | XP\_065052233\_5261-model1.51 | -4.27869 |
|  | XP\_065052233\_5261-model1.52 | -4.27869 |
|  | XP\_065052233\_5261-model1.53 | -4.27851 |
|  | XP\_065052233\_5261-model1.54 | -4.27851 |
|  | XP\_065052233\_5261-model1.55 | -4.27851 |
|  | XP\_065052233\_5261-model1.56 | -4.27851 |
|  | XP\_065052233\_5261-model1.57 | -4.27851 |
|  | XP\_065052233\_5261-model1.58 | -4.27851 |
|  | XP\_065052233\_5261-model1.59 | -4.27851 |
|  | XP\_065052233\_5261-model1.60 | -4.27766 |
|  | XP\_065052233\_5261-model1.61 | -4.27061 |
|  | XP\_065052233\_5261-model1.62 | -4.26594 |
|  | XP\_065052233\_5261-model1.63 | -4.26594 |
|  | XP\_065052233\_5261-model1.64 | -4.26395 |
|  | XP\_065052233\_5261-model1.65 | -4.25752 |
|  | XP\_065052233\_5261-model1.66 | -4.22432 |
|  | XP\_065052233\_5261-model1.67 | -4.21895 |
|  | XP\_065052233\_5261-model1.68 | -4.21488 |
|  | XP\_065052233\_5261-model1.69 | -4.18332 |
|  | XP\_065052233\_5261-model1.70 | -4.18039 |
|  | XP\_065052233\_5261-model1.71 | -4.1608 |
|  | XP\_065052233\_5261-model1.72 | -4.14417 |
|  | XP\_065052233\_5261-model1.73 | -4.13856 |
|  | XP\_065052233\_5261-model1.74 | -4.12675 |
|  | XP\_065052233\_5261-model1.75 | -4.09853 |
|  | XP\_065052233\_5261-model1.76 | -4.03445 |
|  | XP\_065052233\_5261-model1.77 | -4.00004 |
|  | XP\_065052233\_5261-model1.78 | -3.93406 |
|  | XP\_065052233\_5261-model1.79 | -3.913 |
| # cluster 2 - Size: 7 |  |  |
|  | XP\_065052233\_5261-model2 | -4.28663 |
|  | XP\_065052233\_5261-model2.1 | -4.28663 |
|  | XP\_065052233\_5261-model2.2 | -4.25857 |
|  | XP\_065052233\_5261-model2.3 | -4.24544 |
|  | XP\_065052233\_5261-model2.4 | -4.21308 |
|  | XP\_065052233\_5261-model2.5 | -4.16883 |
|  | XP\_065052233\_5261-model2.6 | -4.09747 |
| # cluster 3 - Size: 12 |  |  |
|  | XP\_065052233\_5261-model3 | -4.04814 |
|  | XP\_065052233\_5261-model3.1 | -4.04814 |
|  | XP\_065052233\_5261-model3.2 | -4.03836 |
|  | XP\_065052233\_5261-model3.3 | -4.03836 |
|  | XP\_065052233\_5261-model3.4 | -4.03836 |
|  | XP\_065052233\_5261-model3.5 | -4.02279 |
|  | XP\_065052233\_5261-model3.6 | -4.02279 |
|  | XP\_065052233\_5261-model3.7 | -4.02279 |
|  | XP\_065052233\_5261-model3.8 | -3.99491 |
|  | XP\_065052233\_5261-model3.9 | -3.9715 |
|  | XP\_065052233\_5261-model3.10 | -3.96094 |
|  | XP\_065052233\_5261-model3.11 | -3.96094 |
| # cluster 4 - Size: 1 |  |  |
|  | XP\_065052233\_5261-model4 | -3.95997 |
|  |  |  |
